# Supplementary material for: Association between dietary contribution of ultra-processed foods and urinary concentrations of phthalates and bisphenol in a nationally representative sample of the US population aged 6 years and older
Source: PLoS One. 2020 Jul 31;15(7):e0236738. doi: 10.1371/journal.pone.0236738 (PMC7394369; doi:10.1371/journal.pone.0236738)
Supplement: Attachment — Submitted filename: PONE-D-20-02900_sgs.pdf [file pone.0236738.s011.pdf]

PLOS ONE

Association between dietary share of ultra-processed foods and urinary concentrations of phthalates and bisphenol in a nationally representative US sample

--Manuscript Draft--

**Manuscript Number:**	PONE-D-20-02900
**Article Type:**	Research Article
**Full Title:**	Association between dietary share of ultra-processed foods and urinary concentrations of phthalates and bisphenol in a nationally representative US sample
**Short Title:**	Dietary share of ultra-processed foods and urinary concentrations of phthalates and bisphenol in the US
**Corresponding Author:**	Euridice Martinez Steele<br>Universidade de Sao Paulo Faculdade de Saude Publica<br>Sao Paulo, Sao Paulo BRAZIL
**Keywords:**	NHANES; NOVA; ultra-processed foods; phthalates; bisphenol
**Abstract:**	Ultra-processed food consumption has been associated with several health outcomes such as obesity, hypertension, cardiovascular disease or cancer. The adverse nutritional composition of these products, and the presence of food additives, neoformed contaminants and contact materials such as phthalates and bisphenol may be some of the potential pathways through which ultra-processed food influences disease outcomes. The aim of this study was to examine the association between dietary contribution of ultra-processed foods and urinary concentrations of Di(2-ethylhexyl) phthalate (DEHP), Di-isononyl phthalate (DiNP), Di-isodecyl phthalate (DiDP), Di-n-octyl phthalate (DOP/DnOP), Benzylbutyl phthalate (BBzP), Bisphenol A (BPA), F (BPF) and S (BPS) in the US.<br>Participants from cross-sectional 2009-2016 National Health and Nutrition Examination Survey aged 6+ years, with urinary measures and with one 24-hour dietary recall were evaluated. Ultra-processed foods were identified based on the NOVA classification system, a four-group food classification based on the extent and purpose of industrial food processing.<br>Linear regression was used to compare average urinary concentrations (normalized by creatinine-standardized concentrations) across quintiles of energy share of ultra-processed foods. Models incorporated survey sample weights and were adjusted for different sociodemographic and life-style variables. Adjusted geometric means of ΣDiNP, mCNP, mCPP, mBzP and BPF increased monotonically from the lowest to the highest quintile of ultra-processed food consumption. As both phthalates/bisphenol and ultra-processed foods have been previously associated with insulin resistance, diabetes, general/abdominal obesity and hypertension, our results suggest the possibility of contact materials in ultra-processed foods as one link between ultra-processed food and these health outcomes. Future studies could further explore these mechanisms of action.
**Order of Authors:**	Euridice Martinez Steele<br>Neha Khandpur<br>Maria Laura da Costa Louzada<br>Carlos Augusto Monteiro
**Additional Information:**	
**Question**	**Response**
**Financial Disclosure**<br><br>Enter a financial disclosure statement that describes the sources of funding for the work included in this submission. Review the submission guidelines for detailed	EMS- 2018/17972-9 (FAPESP); CAM- 2015/14900-9 (FAPESP)

requirements. View published research articles from *PLOS ONE* for specific examples.<br><br>This statement is required for submission and **will appear in the published article** if the submission is accepted. Please make sure it is accurate.<br><br>**Unfunded studies**<br>Enter: *The author(s) received no specific funding for this work.*<br><br>**Funded studies**<br>Enter a statement with the following details:<br>• Initials of the authors who received each award<br>• Grant numbers awarded to each author<br>• The full name of each funder<br>• URL of each funder website<br>• Did the sponsors or funders play any role in the study design, data collection and analysis, decision to publish, or preparation of the manuscript?<br>• **NO** - Include this sentence at the end of your statement: *The funders had no role in study design, data collection and analysis, decision to publish, or preparation of the manuscript.*<br>• **YES** - Specify the role(s) played.<br><br>* typeset	
**Competing Interests**<br><br>Use the instructions below to enter a competing interest statement for this submission. On behalf of all authors, disclose any competing interests that could be perceived to bias this work—acknowledging all financial support and any other relevant financial or non-financial competing interests.<br><br>This statement **will appear in the published article** if the submission is accepted. Please make sure it is accurate. View published research articles from *PLOS ONE* for specific examples.	The authors have declared that no competing interests exist.

**NO authors have competing interests**<br><br>Enter: *The authors have declared that no competing interests exist.*<br><br>**Authors with competing interests**<br><br>Enter competing interest details beginning with this statement:<br><br>*I have read the journal's policy and the authors of this manuscript have the following competing interests: [insert competing interests here]*<br><br>* typeset	
**Ethics Statement**<br><br>Enter an ethics statement for this submission. This statement is required if the study involved:<br><br>• Human participants<br>• Human specimens or tissue<br>• Vertebrate animals or cephalopods<br>• Vertebrate embryos or tissues<br>• Field research<br><br>Write "N/A" if the submission does not require an ethics statement.<br><br>General guidance is provided below. Consult the submission guidelines for detailed instructions. **Make sure that all information entered here is included in the Methods section of the manuscript.**	The National Center for Health Statistics Research Ethics Review Board approved the study protocol. All participants provided written informed consent; parents or guardians provided consent for participants < 18 years of age.

Powered by Editorial Manager® and ProduXion Manager® from Aries Systems Corporation

**Format for specific study types**<br><br>**Human Subject Research (involving human participants and/or tissue)**<br>• Give the name of the institutional review board or ethics committee that approved the study<br>• Include the approval number and/or a statement indicating approval of this research<br>• Indicate the form of consent obtained (written/oral) or the reason that consent was not obtained (e.g. the data were analyzed anonymously)<br><br>**Animal Research (involving vertebrate animals, embryos or tissues)**<br>• Provide the name of the Institutional Animal Care and Use Committee (IACUC) or other relevant ethics board that reviewed the study protocol, and indicate whether they approved this research or granted a formal waiver of ethical approval<br>• Include an approval number if one was obtained<br>• If the study involved *non-human primates*, add *additional details* about animal welfare and steps taken to ameliorate suffering<br>• If anesthesia, euthanasia, or any kind of animal sacrifice is part of the study, include briefly which substances and/or methods were applied<br><br>**Field Research**<br><br>Include the following details if this study involves the collection of plant, animal, or other materials from a natural setting:<br>• Field permit number<br>• Name of the institution or relevant body that granted permission	
**Data Availability**<br><br>Authors are required to make all data underlying the findings described fully available, without restriction, and from the time of publication. PLOS allows rare exceptions to address legal and ethical concerns. See the PLOS Data Policy and FAQ for detailed information.	Yes - all data are fully available without restriction

A Data Availability Statement describing where the data can be found is required at submission. Your answers to this question constitute the Data Availability Statement and **will be published in the article**, if accepted.<br><br>**Important:** Stating 'data available on request from the author' is not sufficient. If your data are only available upon request, select 'No' for the first question and explain your exceptional situation in the text box.<br><br>Do the authors confirm that all data underlying the findings described in their manuscript are fully available without restriction?	
**Describe where the data may be found in full sentences. If you are copying our sample text, replace any instances of XXX with the appropriate details.**<br><br>• If the data are **held or will be held in a public repository**, include URLs, accession numbers or DOIs. If this information will only be available after acceptance, indicate this by ticking the box below. For example: *All XXX files are available from the XXX database (accession number(s) XXX, XXX.*).<br>• If the data are all contained **within the manuscript and/or Supporting Information files**, enter the following: *All relevant data are within the manuscript and its Supporting Information files.*<br>• If neither of these applies but you are able to provide **details of access elsewhere**, with or without limitations, please do so. For example:<br><br>*Data cannot be shared publicly because of [XXX]. Data are available from the XXX Institutional Data Access / Ethics Committee (contact via XXX) for researchers who meet the criteria for access to confidential data.*<br><br>*The data underlying the results presented in the study are available from (include the name of the third party*	All analyses used publicly available datasets downloadable from NHANES website (<https://wwwn.cdc.gov/nchs/nhanes/Default.aspx>).

and contact information or URL).<br>• This text is appropriate if the data are owned by a third party and authors do not have permission to share the data.<br><br>* typeset	
Additional data availability information:	

Powered by Editorial Manager® and ProduXion Manager® from Aries Systems Corporation

Manuscript Click here to download Manuscript Phthalates_2009-16_20200131.docx

Title: Association between dietary share of ultra-processed foods and urinary concentrations of phthalates and bisphenol in a nationally representative US sample

Authors: Eurídice Martínez Steele1,2; Neha Khandpur1,2,3; Maria Laura da Costa Louzada1,2; and Carlos A. Monteiro1,2.

[1]Department of Nutrition, School of Public Health, University of São Paulo, São Paulo, Brazil;

[2]Center for Epidemiological Studies in Health and Nutrition, University of São Paulo, São Paulo, Brazil;

[3]Department of Nutrition, Harvard T.H. Chan School of Public Health, Boston, USA

Corresponding author: Eurídice Martínez Steele, Departamento de Nutrição, Faculdade de Saúde Pública, Universidade de São Paulo, Av. Dr. Arnaldo, 715, São Paulo 01246-907, Brazil. E-mail: emar_steele@hotmail.com

Highlights:

- Ultra-processed food consumption has been associated with several health outcomes.
- Presence of phthalates and bisphenol may be potential links.
- ΣDiNP, mCNP, mCPP, mBzP and BPF increased with ultra-processed food consumption.

Abbreviations:

- Di(2-ethylhexyl) phthalate (DEHP)
- Di-isononyl phthalate (DiNP)
- Di-isodecyl phthalate (DiDP)
- Di-n-octyl phthalate (DOP/DnOP)
- Benzylbutyl phthalate (BBzP)
- Bisphenol A (BPA)
- Bisphenol F (BPF)
- Bisphenol S (BPS)

Abstract

Ultra-processed food consumption has been associated with several health outcomes such as obesity, hypertension, cardiovascular disease or cancer. The adverse nutritional composition of these products, and the presence of food additives, neoformed contaminants and contact materials such as phthalates and bisphenol may be some of the potential pathways through which ultra-processed food influences disease outcomes. The aim of this study was to examine the association between dietary contribution of ultra-processed foods and urinary concentrations of Di(2-ethylhexyl) phthalate (DEHP), Di-isononyl phthalate (DiNP), Di-isodecyl phthalate (DiDP), Di-n-octyl phthalate (DOP/DnOP), Benzylbutyl phthalate (BBzP), Bisphenol A (BPA), F (BPF) and S (BPS) in the US.

Participants from cross-sectional 2009-2016 National Health and Nutrition Examination Survey aged 6+ years, with urinary measures and with one 24-hour dietary recall were evaluated. Ultra-processed foods were identified based on the NOVA classification system, a four-group food classification based on the extent and purpose of industrial food processing.

Linear regression was used to compare average urinary concentrations (normalized by creatinine-standardized concentrations) across quintiles of energy share of ultra-processed foods. Models incorporated survey sample weights and were adjusted for different sociodemographic and life-style variables. Adjusted geometric means of ΣDiNP, mCNP, mCPP, mBzP and BPF increased monotonically from the lowest to the highest quintile of ultra-processed food consumption. As both phthalates/bisphenol and ultra-processed foods have been previously associated with insulin resistance, diabetes, general/abdominal obesity and hypertension, our results suggest the possibility of contact materials in ultra-processed foods as one link between ultra-processed food and these health outcomes. Future studies could further explore these mechanisms of action.

Keywords: NHANES; NOVA; ultra-processed foods; phthalates; bisphenol

1. Introduction

Ultra-processed foods are defined by NOVA (not an acronym) classification, as industrial formulations of food-derived substances (such as oils, fats, sugars, starch, protein isolates) that contain little or no whole food and often include flavorings, colorings, emulsifiers and other cosmetic additives [1]. During the past decades the consumption of ultra-processed foods has increased worldwide [2-8]. Prospective studies have linked ultra-processed food intake with a higher risk of overweight, obesity [9; 10], hypertension [11], dyslipidaemia [12], overall and breast cancer [13], cardiovascular diseases [14], diabetes [15] and all- cause mortality [16-18].

Several mechanisms may potentially explain these associations. Ultra-processed foods have a higher content in total fat, saturated fat, added sugar, energy density, and salt, together with a lower fibre, vitamin and mineral density, as compared to non-ultra-processed foods, and their consumption result in overall deterioration of the nutritional quality of the diet [1; 19]. Their convenience and hyperpalatability simultaneously lower consumption of healthy non-ultra-processed foods such as fruit and vegetables [19]. Ultra-processed foods may also affect glycaemic responses and satiety [20] and create a gut environment that selects microbes that promote inflammatory disease [21]. Cosmetic additives frequently added to ultra-processed foods (such as glutamates, emulsifiers, sulfites and carrageenan) or several compounds that are neoformed during their processing (such as acrylamide or acrolein) could also play a role [14]. A recent inpatient ad libitum cross-over randomized controlled trial conducted by the US National Institute of Health concluded that individuals consumed 508 more kcal and gained an average of 0.8 kg of weight during the ultra-processed diet (> 80% energy from ultra-processed foods) and lost 0.9 kg during the non-ultra-processed diet. The fact that diets were matched for presented total calories, macronutrients and fiber, suggests that mechanisms other than the dietary nutrient profile such as quicker eating time or reduced signs of satiety might explain these results [22].

While not directly related with the food per se, the packaging of ultra-processed foods might also help explain the health effects of these products [14]. Ultra-processed foods are frequently packaged in materials that are sources of endocrine disrupting chemicals such as phthalates and bisphenol, associated with adverse health outcomes especially in pregnancy [23; 24]. A large body of cross-sectional studies have specifically linked Bisphenol A (BPA) exposure with higher risk of diabetes, general/abdominal obesity and hypertension [25] and phthalates with diabetes [26] and insulin resistance [27].

Though contained in many consumer products, food is a ubiquitous source of phthalates and bisphenols attributed mainly to food production, processing, and packaging practices; food storage conditions and, also animal feeding practices. These non- chemically bond to the polymeric matrix chemicals, are known to migrate from food contact materials (plastics, paper, metal, glass, and printing inks) that protect food from physical damage and microbial spoilage [24]. The long-shelf life and ready-to-eat characteristics of ultra-processed foods entails that these substances are likely to leach into the food product, making ultra-processed foods a potential delivery vehicle for phthalates and bisphenols in humans. This leakage could be more severe in ready-to-eat foods served in paper and cardboard containers used for take-away food [28] or heated [24; 29] or even served warm in plastic containers.

The objective of our study was to examine the association between dietary contribution of ultra-processed food and exposure to Di(2-ethylhexyl) (ΣDEHP), Di-isononyl (ΣDiNP), Monocarboxynonyl (mCNP), Mono(3-carboxypropyl) (mCPP) and Monobenzyl (mBzP) phthalates, and Bisphenol A, F and S (BPA, BPF and BPS, respectively) in a US population aged 6 years and older.

Very few studies have explored this topic. To our knowledge, only one other study has assessed the link between ultra-processed food consumption and phthalates/bisphenol [30]. The authors of this study found a positive association between ultra-processed foods and urinary concentrations of Monocarboxynonyl (mCNP), Mono(3-carboxypropyl) (mCPP), and mono-(carboxyisoctyl) (MCOP) but not mono-benzyl (MBzP), Di(2-ethylhexyl) (ΣDEHP), or bisphenols. This study included data from NHANES cycle 2013-14 and was most likely underpowered to detect associations. The current study addresses this gap by including data from 2009 to 2016. We also examined the departure from linear relationship between percent of calorie from ultra-processed foods and urinary metabolite concentrations and carried out several sensitivity analyses to test the robustness of associations.

2. Material and Methods

2.1 Data source, population and sampling

We used nationally representative data from National Health and Nutrition Examination Survey (NHANES) 2009-2016 (four 2-year cycles). NHANES is a continuous, nationally representative, cross-sectional survey of the non-institutionalized, civilian US residents conducted by The Centers for Disease Control and Prevention [31]. Participants were recruited using a four-stage sample design based on the selection of counties, blocks, households, and the number of people within households.

The survey included an interview conducted in the home and a subsequent health examination was performed at a mobile examination center (MEC) that included blood and urine collection. All NHANES participants who were examined at MECs were eligible for two 24-hour dietary recall interviews: the first one collected in-person in the MEC [32] and the second by telephone, 3 to 10 days later [33]. Dietary interviews were conducted by trained interviewers using the validated [34-36] US Department of Agriculture Automated Multiple-Pass Method [37]. Proxy-assisted interviews were conducted with children 6–11 years old; and participants ≥ 12 years old completed the dietary interview for themselves.

Our analytical sample comprised individuals aged 6 years or older (urinary metabolite concentrations were not measured in under 6 year old children), who provided a urine sample for phthalate or bisphenol analysis, completed a 24-hr dietary recall survey and had complete information on all variables of interest, resulting in a final sample size of 9,416 participants for phthalate analysis and 9,420 for Bisphenol A. The final sample size for Bisphenol F and S analyses was 4,655, as these 2 urine metabolites were only measured in cycles 2013-2016 [38] (Table 1).

The National Center for Health Statistics Research Ethics Review Board approved the study protocol. All participants provided written informed consent; parents or guardians provided consent for participants < 18 years of age.

2.2 Urinary metabolite measurement

Due to their quick metabolism and consequent short half-lives (<24 h), exposures to phthalates and bisphenols are best characterized in urine (compared with blood) [39; 40].

Our study focused on phthalate and bisphenol metabolites (expressed in ng/mL) measured in all 4 studied cycles including Mono(2-ethylhexyl) (mEHP), Mono(2-ethyl-5-hydroxyhexyl)

(mEHHP), Mono(2-ethyl-5-oxohexyl) (mEOHP), Mono(2-ethyl-5-carboxypentyl) (mECPP), Mono-isononyl (mNP/miNP), Monocarboxyoctyl (mCOP), Monocarboxynonyl (mCNP), Mono(3-carboxypropyl) (mCPP), Monobenzyl (mBzP), Monoethyl (mEP), Mono-n-butyl (mnBP), Mono-isobutyl (miBP), Bisphenol A (BPA) and its replacements Bisphenol S (BPS) and Bisphenol F (BPF) (Supplementary Table 1).

Urine specimens were collected in spot urine samples at the MEC and processed, stored (under appropriate frozen (–20°C) conditions), and shipped to the Division of Laboratory Sciences, National Center for Environmental Health, Centers for Disease Control and Prevention for analysis.

Briefly, chemical analytes were quantified in urine using solid-phase extraction coupled online with high-performance liquid chromatography and tandem mass spectrometry and expressed as wet weights (ng/mL) [41; 42]. The limits of detection (LOD) ranged from 0.2 to 1.2 ng/mL for the phthalate metabolites [41] and from 0.1 to 0.4 ng/mL for BP [42]. Where LOD varied across study cycles, we assumed the maximal LOD for each phthalate and BP metabolite in our analysis to facilitate aggregation of data across study cycles [43].

For the sample of 9,416, 2858 individuals were below the lower detection limit (LLOD) for mEHP (0.8 ng/mL), 34 individuals for mEHHP (0.4 ng/mL), 40 individuals for mEOHP (0.2 ng/mL), 16 individuals for mECPP (0.4 ng/mL), 5533 for mNP/miNP (0.9 ng/mL), 20 for mCOP (0.3 ng/mL), 121 for mCNP (0.2 ng/mL), 808 for mCPP (0.4 ng/mL), 158 for mBzP (0.3 ng/mL), 18 for mEP (1.2 ng/mL), 200 for mBP/mnBP (0.4 ng/mL) and 130 for miBP (0.8 ng/mL). For the sample of 9,420, 637 individuals were below the LLOD for BPA (0.4 ng/mL). For the sample of 4655, 466 individuals were below the LLOD for BPS (0.1 ng/mL) and 2,093 for BPF (0.2 ng/mL).

In NHANES, urinary phthalate and BP measurements below the limits of detection of the used method were replaced with $1/\sqrt{2}$ fraction of the detection limit.

Individual metabolites (expressed in ng/mL) were rescaled in ηmol/mL by dividing each one by its molar mass. We calculated molar sums, representing classes of chemicals or parent compounds, by summing individual metabolite concentrations [44]: ΣDEHP (sum of di(2-ethylhexyl) phthalate metabolites: MEHP, MEHHP, MEOHP, and MECPP), and ΣDiNP (sum of Di-isononyl phthalate metabolites: mNP/miNP, and mCOP). Even though low molecular weight metabolites (ΣLMWP) are not present in the food supply [24] we also assessed the association with ΣLMWP (sum of low molecular weight metabolites: MEP, MBP, and MiBP) expecting no association.

In order to correct for urine dilution, urinary metabolite concentrations were normalized by urinary creatinine (and expressed in ηmol/g creatinine) [45]. This was done by dividing each individual metabolite concentration value (expressed in nmol/mL) by the corresponding urinary creatinine value (expressed in g/mL). Creatinine was measured using Beckman Synchron CX3 Clinical Analyser at the University of Minnesota [46].

2.3 Food classification according to processing

During the dietary interview, participants were prompted to list all foods and beverages consumed the day prior to the interview (in a 24-hr period, from midnight to midnight). All recorded food items (Food Codes) were classified according to NOVA, a food classification based on the extent and purpose of industrial food processing. NOVA includes 4 groups: “unprocessed or minimally processed foods” (such as fresh, dry or frozen fruits or vegetables; packaged grains and pulses; grits, flakes or flours made from corn, wheat or cassava; pasta, fresh or dry, made from flours and water; eggs; fresh or frozen meat and fish and fresh or pasteurized milk); “processed culinary ingredients” (including sugar, oils, fats, salt, and other substances extracted from foods and used in kitchens to season and cook unprocessed or minimally processed foods and to make culinary preparations), “processed foods” (including canned foods, sugar-coated dry fruits, salted meat products, cheeses and freshly made unpackaged breads, and other ready-to-consume products manufactured with the addition of salt or sugar or other substances of culinary use to unprocessed or minimally processed foods), and “ultra-processed foods”.

The NOVA group of ultra-processed foods of particular interest in this study, includes soft drinks, sweet or savory packaged snacks, confectionery and industrialized desserts, mass-produced packaged breads and buns, poultry and fish nuggets and other reconstituted meat products, instant noodles and soups, and many other ready-to-consume formulations of several ingredients. Besides salt, sugar, oils, and fats, these ingredients include food substances not commonly used in culinary preparations, such as modified starches, hydrogenated oils, protein isolates and classes of additives whose purpose is to imitate sensorial qualities of unprocessed or minimally processed foods and their culinary preparations, or to disguise undesirable qualities of the final product. These additives include colorants, flavorings, non-sugar sweeteners, emulsifiers, humectants, sequestrants, and firming, bulking, de-foaming, anti-caking and glazing agents. Unprocessed or minimally processed foods represent a small proportion of or are even absent from the list of ingredients

of ultra-processed foods. A detailed definition of each NOVA food group and examples of food items classified in each group are shown elsewhere [47].

For all food items (Food Codes) judged to be a handmade recipe, the classification was applied to the underlying ingredients (Standard Reference Codes -SR Codes-) obtained from the USDA Food and Nutrient Database for Dietary Studies (FNDDS) [FNDDS] as further explained in previously published papers [48; 49].

Food items were sorted into mutually exclusive food subgroups within unprocessed or minimally processed foods (n=11), processed culinary ingredients (n=4), processed foods (n=4) and ultra-processed foods (n=18) [48].

Phthalates and Bisphenol may be potentially concentrated in foods served in paper and cardboard containers used for take-away food [28] or heated [24; 29] or even served warm in plastic containers. Thus, for current study ultra-processed food subgroups were sorted into two groups: (1) Ready-to-heat/ Frozen meals (Frozen and shelf-stable plate meals; Pizza; French fries and other potato products; Sandwiches and hamburgers on bun); (2) Other ultra-processed foods (Breads; Cakes, cookies and pies; Salty snacks; Soft drinks, carbonated; Fruit drinks; Breakfast cereals; Sauces, dressings and gravies; Reconstituted meat or fish products; Sweet snacks; Ice cream and ice pops; Milk-based drinks; Desserts; Instant and canned soups; Other ultra-processed food).

2.4 Dietary assessment

USDA's Food and Nutrient Database for Dietary Studies 5.0, 2011-2012, 2013-2014 and 2015-2016 [50] were used to code dietary intake data and calculate Food Code energy and total fat energy intakes (kcal). For handmade recipes, we calculated the underlying ingredient (SR Code) energy and total fat values using variables from both FNDDS databases [50] and USDA National Nutrient Database for Standard Reference, Release 24, 26 and 28 [51].

The dietary recall interview also asked about the source from which each food was obtained. Fast food was defined as food obtained from restaurants without waiter/waitress service, or from pizza restaurants regardless of waiter/ waitress service.

For these analyses, we extracted 24-hr total energy intake, energy intake derived from ultra-processed foods, energy intake derived from total fat, total energy intake derived from fat in ultra-processed foods and total energy intake derived from fast food, by participant. We additionally extracted 24-hr total energy intake derived from both Ready-to-heat and Other ultra-processed food NOVA subgroups.

2.5 Covariates

Potential confounders were identified from the literature [24; 30]. Socio-demographic covariates included sex, age, race/ethnicity, family income and cycle. Age was grouped into three categories (6-11 years, 12-19 years, 20 years of age and over) [31]. Race/ethnicity was categorized as Mexican-American, Other Hispanic, Non-Hispanic White, Non-Hispanic Black and Other Races including Multi-Racial. With respect to family income, ratio of family income to poverty was established and categorized based on Supplemental Nutrition Assistance Program (SNAP) eligibility as 0.00–1.30, >1.30–3.50, and 3.50 and above [31]. Cycle included the following categories: 2009-10, 2011-12, 2013-14 and 2015-16.

BMI was calculated by dividing measured weight by height squared (kg/m2) [52] and used as a categorical covariate (underweight, normoweight, overweight and obesity). Among adults, BMI values of <20 were classified as underweight, ≥25 as overweight and ≥30 kg/m2 as obesity according to World Health Organization criteria [53]. Among children, cutoff criteria were based on the Centers for Disease Control and Prevention's sex-specific 2000 BMI-for-age-sex growth charts for the United States: Underweight (BMI < 5th percentile), Normal weight (BMI 5th to < 85th percentiles), Overweight (BMI 85th to < 95th percentiles) and Obese (BMI ≥ 95th percentile) [54].

Physical activity was categorized into three intensity levels – light (<150 minutes per week of moderate intensity equivalent activity), moderate (150 to 300 minutes per week of moderate-intensity equivalent activity) and vigorous (>300 minutes per week of moderate- intensity equivalent activity) [55]. In individuals 12 + years of age, MET (metabolic equivalent of task)-minutes per week were calculated based on reported frequency and duration of physical activity in a typical week. In children<12 years of age, minutes per week were calculated based on response to question “Days physically active during past week (at least 60 minutes)”.

Smoking status was categorized as current smoker and non-smoker. Energy intake above recommended levels was coded as yes/ no according to sex–age–physical activity levels [56]. Total fat intake (% of total energy intake) was used as continuous or categorized as above >30% (yes/no) [57]. Fat in ultra-processed food derived total energy intake (% of total energy intake) was categorized as above median (18.7%) (yes/no) and into quintiles. Fast food intake (% of total energy intake) was used as continuous.

2.6 Data Analysis

All available day 1 dietary intake data for each participant were utilized.

First, we evaluated the mean dietary contribution of ultra-processed food (% of total energy) and urinary metabolite concentrations, overall and across socio-demographic, life-style and dietary characteristics of respondents using linear regression. Test of linear trend was performed for ordinal variables and Wald test with Bonferroni inequality adjustment for multiple comparisons was used for non-ordinal categorical variables or in the absence of a statistically significant linear trend. As urinary metabolite concentrations (both in ηmol/ ml and standardized in ηmol/g creatinine) had skewed distributions, these variables were log transformed (using natural logarithms) and least squares geometric means were presented.

The average metabolite urinary concentrations were compared across quintiles of the dietary share of ultra-processed food using linear regression models. For each metabolite, four models were explored: 1) crude (in ηmol/ml); 2) standardized in ηmol/g creatinine; 3) standardized in ηmol/g creatinine and adjusted for socio-demographic variables (sex, age group, race/ethnicity, ratio of family income to poverty, cycle); and 4) standardized in ηmol/g creatinine and adjusted for socio-demographic + energy intake above recommended (yes, no), BMI (categorical), physical activity (categorical) and current smoking (yes, no[illegible]rom these regression models, we estimated: a) least squares geometric means of urinary chemical concentrations across quintiles of ultra-processed food consumption as $e^{(\text{least squares means})}$. On the basis of the multivariable regression models, we calculated and plotted the estimated margins for each metabolite according to quintile of relative ultra-processed food consumption[illegible] Margins were estimated at the means of all covariates; b) percent difference in urinary chemical concentrations comparing first and fifth quintile of ultra-processed food consumption as $(e^{(\beta)} – 1) \times 100\%$ with 95% CIs estimated as $(e^{(\beta \pm \text{critical value} \times \text{SE})} – 1)$, where β and SE are the estimated regression coefficient and standard error for the fifth quintile, respectively. Tests of linear trend were also performed to evaluate the effect of quintiles as a single continuous variable.

Thereafter, we used the restricted cubic spline in the multivariable linear regression models with five knots (5th, 27.5th, 50th, 72.5th, and 95th) following Harrell´s recommendations [58] to examine the shape of the dose-response relationship curve between percent of calorie from ultra-processed foods and urinary metabolite concentrations [59].

To test the robustness of the associations, the following sensitivity tests were performed:

1. Previous studies have suggested that foods high in fat may be more contaminated by phthalates and BPA that are more lipophilic [60; 61]. For this reason and to test the robustness of the associations, we conducted sensitivity analyses (using the

multivariable socio-demographic and life-style adjusted model) also adjusting for total fat intake (% of total energy intake) (continuous). [illegible] further test this, we also carried out fully adjusted secondary analysis using as exposure variable the quintiles of fat in ultra-processed food derived total energy intake (% of total energy intake).

2. As studies have suggested that fast food may be a unique dietary source of ΣDiNP and mBzP [62; 63], we carried out additional sensitivity analysis also adjusting for fast food intake (% of total energy intake).
3. Because of lack of consensus on the most appropriate method to adjust for urinary dilution, the use of different methods for urinary dilution adjustment has been recommended [40]. For this reason, sensitivity analyses were also carried out adjusting for creatinine concentration (milligrams per deciliter) while using crude metabolite measures (ηmol/mL) as suggested by Barr et al. [64].

Effect modification by sex, age group and data collection cycle were tested by including a multiplicative interaction term (tested both as continuous and as dummy variable) in the multivariable socio-demographic and life-style adjusted model. Analyses were stratified according to statistically significant interaction variables.

We also examined the association between dietary share of Ready-to-heat and Other ultra-processed food (each categorized into tertiles) and Phthalate/Bisphenol levels using adjusted models.

Finally, we performed secondary analyses to test the association between quintiles of dietary contribution of each of the remaining three NOVA groups (minimally processed foods, processed culinary ingredients and processed foods) and urinary concentrations.

NHANES survey sample weights were used in all analyses to account for differential probabilities of selection for the individual domains, nonresponse to survey instruments, and differences between the final sample and the total US population. The Taylor series linearization variance approximation procedure was used for variance estimation in all analysis to account for the complex sample design and the sample weights [31].

Because we combined four survey cycles, new sample weights were calculated for each participant according to the analytical guidelines [31].

Statistical hypotheses were tested using a two-tailed $p<0.05$ level of significance. Data were analyzed using Stata statistical software package version 14.

3. Results

Overall dietary contribution of ultra-processed foods was 58% and decreased with age and cycle, was higher among non-Hispanic white and black (and lower among other race) and was lower among the highest income level. Ultra-processed food consumption varied according to BMI status and was higher among smokers, and among individuals with energy and total fat intake above recommended (Table 2).

As can be seen in table 2, Phthalate/Bisphenol concentration levels were higher among women (except for BPS), decreased with age (except BPF which did not change and BPS which increased with age) and cycle (except for BPS) and varied across race/ethnicities (except for BPA) and income levels (except for BPF). Concentration levels of some metabolites also varied according to BMI status (decreased with BMI for ΣDEHP, mCPP and mBzP), physical activity (higher among middle physical activity level for ΣDEHP, ΣDiNP, mCNP and mCPP) and smoking status (higher among smokers for BPA, BPF, BPS and mBzP, and higher among non-smokers in remaining metabolites). For some metabolites, concentration levels varied according to total energy intake above recommended levels (positive association for ΣDEHP, mCNP and mCPP) and total fat above recommended levels (positive association for ΣDiNP and mCNP, and inverse for BPS), and fat in ultra-processed foods derived total energy intake (positive association for all metabolites, except for BPS which was inverse and ΣDEHP with no associatio[illegible]

Fully adjusted models showed a positive association between ultra-processed food quintiles and ΣDiNP, mCNP, mCPP, mBzP, and BPF concentration levels. Conversely, a lack of association was observed for ΣDEHP and BPA, and an inverse association for BPS (Table 3). Compared to the lowest ultra-processed food consumers (first quintile), the highest quintile had 23.4% (95% CI: 7.9% to 41.2%) higher levels of mBzP, 14.6% (95% CI: 4.4% to 25.8%) higher levels of mCNP, 11.5% (95% CI: 0.2% to 24.1%) higher levels of mCPP, 10.7% (95% CI: -0.6% to 23.3%) higher levels of mBzP, 6.2% (95% CI: -2.7% to 15.9%) higher levels of BPA, and 33.8% (95% CI: 11.7% to 60.3%) higher levels of BPF. On the other hand, the highest quintile of ultra-processed food had 25.1% (95% CI: 12.0 to 36.2%) lower levels of BPS.

No association (p for linear trend=0.154) was observed between ultra-processed food consumption and ΣLMWP (data not shown).

Dose-response curve between dietary contribution of ultra-processed food and urinary metabolite concentrations using restricted cubic splines are displayed in Supplementary Figure

1. There was evidence of a dose-response association with no departure from linearity ($p>0.05$ for linearity) for mCPP and ΣDiNP.

In sensitivity analyses, additional adjustment for total fat intake did not change the main effects, though the positive association with mCPP became not statistically significant (Supplementary Table 2). When adjusting for fast food intake, the association with ΣDiNP and mCPP became non-significant. Further adjustment for creatinine concentration (as covariate), did not change the main effects though the association with BPA became significant.

The strength of the association with urinary metabolites remained virtually the same when using quintiles of fat in ultra-processed food derived total energy intake (% of total energy intake) except for mBzP which became non-significant (Supplementary Table 3).

The association between ultra-processed food and urinary metabolites were not modified by cycle, age or sex, except ΣDiNP and BPA. The association of ultra-processed food intake with ΣDiNP was stronger in children than in adults and did not reach statistical significance among adolescents (p for interaction=0.08). A positive non-significant association between ultra-processed food and BPA was found in both men and women, though the association was stronger in men (p for interaction = 0.02) (Supplementary Table 4).

We observed a monotonic increase of ΣDiNP, mCNP, mCPP, mBzP (p for trend ≤ 0.001) and BPA (p for trend = 0.042) with tertiles of Ready-to-heat ultra-processed foods. Non-Ready-to-heat ultra-processed foods were positively associated with BPF concentration (p for trend = 0.005) and inversely associated with BPS (p for trend ≤ 0.001) (Table 4).

4. Discussion

In this cross-sectional study of US population aged 6 + years, there was evidence of monotonic dose-response association between ultra-processed food consumption and urinary concentration of ΣDiNP, mCNP, mCPP, mBzP and BPF concentration levels. No association was observed with ΣDEHP, BPA and an inverse association was observed with BPS. These associations were largely consistent across cycles, age and sex subpopulations, and remained significant after adjusting for total fat intake. A previous study restricted to data from NHANES 2013-14 also reported a positive association between ultra-processed food consumption and mCNP, mCPP and Mono-(carboxyisoctyl) phthalate and a lack of association with ∑DEHP and BPA, however failed to observe a significant positive association with MBzP or BPF or an inverse association with BPS [30].

As expected, no association was observed between ultra-processed food consumption and ∑LMWP, which are primarily used in personal care products and cosmetics [24].

The lack of association between ∑DEHP and ultra-processed food was also observed with quintiles of minimally processed foods and processed foods (Supplementary Tables 5, 7). However, we did find an association with quintiles of processed culinary ingredients (Supplementary Table 6), which may be explained by the lipophilic nature of ∑DEHP which tend to concentrate in fattier foods such as butter, cream, cooking oils and animal fats (processed culinary ingredients) [60; 65].

While a positive though non-significant association was observed between BPA and both ultra-processed foods and processed foods (Supplementary Table 7), we found an inverse association with quintiles of minimally processed foods (Supplementary Table 5), and a lack of association with processed culinary ingredients (Supplementary Table 6). These findings may be explained by the fact that canned food which are mainly processed and ultra-processed foods, are considered the predominant source of BPA [66]. Indeed, contamination of food with BPA is usually caused by contact with food packaging materials containing epoxy resins and PC. Epoxy resins are often used as internal coatings of cans to protect from rusting and corrosion and to prevent direct contact of food with metal can walls, and in metal lids for in glass food jars. BPA in PC containers and coatings can migrate into foods, during storage and processing at elevated temperatures [66; 67].

Due to concerns regarding the health effects of BPA, industries have sought for alternatives such as BPF and BPS [68]. In this study we observed a positive association between ultra-processed food consumption and BPF levels but a negative association with BPS concentration.

In recent years, BPS has been used as a substitute for BPA in thermal papers, while high levels of BPS and low levels of BPA have been found in thermal register receipts. What is still unknown, however, is whether BPS has been used as a substitute for BPA in can coatings [68]. In at least one study, BPS was not detected in any of the canned food composite samples and was detected instead in samples prepared from meat and meat products, indicating that sources of BPS other than can coatings are possible [68].

Epidemiological evidence on food sources of ΣDiNP, mCNP and mBzP is scarce [60; 69]. In our study, ΣDiNP, mCNP and mBzP were positively associated with ultra-processed food consumption. Some studies have suggested that fast food consumption may be a unique source of ΣDiNP [62; 63] and mBzP exposure [63]. Interestingly, when we further adjusted for dietary contribution of fast food, the association between ultra-processed food and ΣDiNP loss statistical significance but not the association with mBzP.

There is some epidemiological evidence of association between consumption of meats and fatty foods such as dairy and MCPP levels [60]. Consistent with these results, we observed a positive association between ultra-processed food consumption and MCPP concentrations which became non-significant with further adjustment by total fat intake or fast food intake.

Our study further suggests that ΣDiNP, mCNP, mCPP, mBzP and BPA may be more concentrated in ultra-processed foods served in paper and cardboard containers used for take away food or potentially heated or maybe served warm in plastic containers, while non-ready-to-heat ultra-processed foods were directly associated with BPF and inversely associated with BPS. Though little is known about the migration of phthalates and bisphenol from food packaging during heating, at least one study observed correlation between migration of dibutyl phthalate (DBP) and heating time [29]. Another study concluded that paper and cardboard used in food packaging may contribute to the inadvertent exposure of consumers to endocrine-disrupting chemicals [28].

We observed that the association of ultra-processed food intake with ΣDiNP was stronger in children than in adults and did not reach statistical significance among adolescents. Similarly, Buckley reported a stronger association between ultra-processed food intake and MCOP among children as compared with adults or adolescents [30]. Differences in types of ultra-processed foods consumed or metabolism between age groups may explain these results.

In our study we observed a positive association between ultra-processed food and urinary concentration of most phthalates and bisphenol, suggesting that contamination by contact materials may be an alternativ[illegible]thway to explain the associations seen between ultra-

processed food and various health outcomes, as previously suggested by Srour [14]. Indeed, BPA exposure has been linked with higher risk of diabetes, general/abdominal obesity and hypertension [25] and phthalates with diabetes [26] and insulin resistance [27].

This study has several strengths. A large, nationally representative sample of the US population was used, increasing generalizability. The disaggregation of recipes into underlying ingredients enabled the calculation of more precise estimates of dietary contribution of ultra-processed foods.

Potential limitations should also be considered. Cross-sectional design of NHANES does not allow the inference of causal relationship between ultra-processed food consumption and urinary metabolite concentrations. However, given the short biologic half-lives (<24 hour) of both phthalates and bisphenol [39], both urine samples and dietary information represent exposures during approximately the same 24-hour period. While multiple or 24-h urine samples are the ideal, reliance on a single spot urine sample corresponds well with short elimination half-lives [40].

Self-reported dietary data are prone to information bias even though 24-hour recalls are the least-biased self-report instrument available [70]. Also, standardized methods and approach of NHANES have been shown to produce accurate intake estimates [34-36] and will therefore be suitable for assessing population averages. The use of dietary contribution of ultra-processed food as exposure should reduce bias introduced by non-differential calorie misreporting from all foods. Differential underreporting of ultra-processed food consumption driven by social desirability bias could lead to underestimation of ultra-processed food dietary contribution or dilute the association between ultra-processed food consumption and urinary concentrations. Although NHANES collects limited information indicative of food processing (i.e. place of meals, product brands), these data are not consistently determined for all food items and may also not provide updated, market representative nutrient information [71], which could lead to modest over or underestimation of the dietary contribution of ultra-processed foods. Lastly, the observed associations may be influenced by residual confounders such as source of food (i.e. food away from home, fast food or vending machine), exposure to materials in contact with foodstuffs, oral contact materials other than food (i.e. toys), dermal contact, dust in the environment or occupational exposure [66].

In conclusion, our findings suggest that ultra-processed food consumption may be a source of exposure to ΣDiNP, mCNP, mCPP, mBzP and BPF in the US population. Future studies should seek to confirm our findings and extend the research to examine health outcomes. As both

phthalates/bisphenol and ultra-processed food have been previously linked with insulin resistance, diabetes, general/abdominal obesity and hypertension, future longitudinal studies may help to better understand the mediating role of contact materials in the association between ultra-processed food consumption and these outcomes.

Acknowledgements: NAP

Funding:

This research received funding from Fundação de Amparo à Pesquisa do Estado de São Paulo (Processo nº 2015/14900-9) and from Fundação de Amparo à Pesquisa do Estado de São Paulo (Processo FAPESP nº 2018/17972-9).

Conflicts of Interest:

The authors declare no conflict of interest.

Author contributions:

EMS and CAM conceived and designed the study including statistical analysis;

EMS performed the statistical analyses;

EMS, NK and CAM analyzed and interpreted the data;

EMS took the lead in writing the manuscript;

EMS, NK, ML and CAM revised the manuscript for important intellectual content.

All authors approved the final manuscript and take full responsibility for the final content.

References

1. Monteiro CA, Cannon G, Levy RB, Moubarac JC, Louzada ML, Rauber F, Khandpur N, Cediel G, Neri D, Martinez-Steele E, Baraldi LG, Jaime PC. Ultra-processed foods: what they are and how to identify them. Public Health Nutr. 2019 Apr;22(5):936-941. doi: 10.1017/S1368980018003762. Epub 2019 Feb 12.
2. Monteiro CA, Moubarac JC, Cannon G, Ng SW, Popkin B. Ultraprocessed products are becoming dominant in the global food system. Obes Rev 2013;14(Suppl 2):21-8. doi:10.1111/obr.12107
3. Moodie R, Stuckler D, Monteiro C, et al, Lancet NCD Action Group. Profits and pandemics: prevention of harmful effects of tobacco, alcohol, and ultra-processed food and drink industries. Lancet 2013; 381:670-9. doi:10.1016/S0140-6736(12)62089-3
4. Moubarac JC, Batal M, Martins AP, et al. Processed and ultraprocessed food products: consumption trends in Canada from 1938 to 2011. Can J Diet Pract Res 2014; 75:15-21. doi:10.3148/75.1.2014.15
5. Martins AP, Levy RB, Claro RM, Moubarac JC, Monteiro CA. Increased contribution of ultra-processed food products in the Brazilian diet (1987-2009). Rev Saude Publica 2013; 47:656-65. doi:10.1590/S0034-8910.2013047004968
6. Juul F, Hemmingsson E. Trends in consumption of ultra-processed foods and obesity in Sweden between 1960 and 2010. Public Health Nutr 2015; 18:3096-107. doi:10.1017/S1368980015000506
7. PAHO. Ultra-processed food and drink products in Latin America: Trends, impact on obesity, policy implications. 2015. http://iris.paho.org/xmlui/bitstream/handle/123456789/7699/9789275118641_eng.pdf?sequence=5&isAllowed=y&ua=1
8. Vandevijvere S, Jaacks LM, Monteiro CA, Moubarac JC, Girling-Butcher M, Lee AC, Pan A, Bentham J, Swinburn B. Global trends in ultraprocessed food and drink product sales and their association with adult body mass index trajectories. Obes Rev. 2019 May 17. doi: 10.1111/obr.12860
9. Mendonça RD, Pimenta AM, Gea A, et al. Ultraprocessed food consumption and risk of overweight and obesity: the University of Navarra Follow-Up (SUN) cohort study. Am J Clin Nutr 2016; 104:1433-40. doi:10.3945/ajcn.116.135004
10. Canhada et al. Ultra-processed foods, incident overweight and obesity, and longitudinal changes in weight and waist circumference: the Brazilian Longitudinal Study of Adult Health (ELSA-Brasil). PHN 2019
11. Mendonça RD, Lopes AC, Pimenta AM, Gea A, Martinez-Gonzalez MA, Bes-Rastrollo M. Ultra-Processed Food Consumption and the Incidence of Hypertension in a Mediterranean Cohort: The Seguimiento Universidad de Navarra Project. Am J Hypertens 2017; 30:358-66.
12. Rauber F, Campagnolo PD, Hoffman DJ, Vitolo MR. Consumption of ultra-processed food products and its effects on children's lipid profiles: a longitudinal study. Nutr Metab Cardiovasc Dis 2015; 25:116-22. doi:10.1016/ j.numecd.2014.08.001
13. Fiolet T, Srour B, Sellem L, et al. Consumption of ultra-processed foods and cancer risk: results from NutriNet-Santé prospective cohort. BMJ 2018; 360:k322. doi:10.1136/bmj.k322
14. Srour B, Fezeu LK, Kesse-Guyot E, Allès B, Méjean C, Andrianasolo RM, Chazelas E, Deschasaux M, Hercberg S, Galan P, Monteiro CA, Julia C, Touvier M. Ultra-processed

food intake and risk of cardiovascular disease: prospective cohort study (NutriNet-Santé). BMJ. 2019 May 29; 365:l1451. doi: 10.1136/bmj.l1451.

15. Bernard Srour, Léopold K. Fezeu, Emmanuelle Kesse-Guyot, Benjamin Allès, Charlotte Debras, Nathalie Druesne-Pecollo, Eloi Chazelas, Mélanie Deschasaux, Serge Hercberg, Pilar Galan, Carlos A. Monteiro, Chantal Julia, Mathilde Touvier. Ultra-processed food consumption and risk of type 2 diabetes 1 among participants of the NutriNet-Santé prospective Cohort (accepted for publication)
16. Rico-Campà A, Martínez-González MA, Alvarez-Alvarez I, Mendonça RD, de la Fuente-Arrillaga C, Gómez-Donoso C, Bes-Rastrollo M. Association between consumption of ultra-processed foods and all cause mortality: SUN prospective cohort study. BMJ. 2019 May 29; 365:l1949. doi: 10.1136/bmj.l1949.
17. Kim H, Hu EA, Rebholz CM. Ultra-processed food intake and mortality in the USA: results from the Third National Health and Nutrition Examination Survey (NHANES III, 1988-1994). Public Health Nutr. 2019 Jul;22(10):1777-1785. doi: 10.1017/S1368980018003890. Epub 2019 Feb 21.
18. Blanco-Rojo R, Sandoval-Insausti H, López-Garcia E, Graciani A, JM Ordovás, Banegas JR, Rodríguez-Artalejo F, Guallar-Castillón P. Consumption of Ultra-Processed Foods and Mortality: A National Prospective Cohort in Spain. Mayo Clin Proc. 2019; 94(11): 2178-2188.
19. Monteiro, C.A., Cannon, G., Lawrence, M., Costa Louzada, M.L. and Pereira Machado, P. 2019. Ultra-processed foods, diet quality, and health using the NOVA classification system. Rome, FAO.
20. Fardet A (2016) Minimally processed foods are more satiating and less hyperglycemic than ultra-processed foods: a preliminary study with 98 ready-to-eat foods. Food Funct 7, 2338–2346.
21. Zinöcker MK & Lindseth IA (2018) The Western diet– microbiome–host interaction and its role in metabolic disease. Nutrients 10, E365.
22. Hall KD, Ayuketah A, Brychta R, Cai H, Cassimatis T, Chen KY, Chung ST, Costa E, Courville A, Darcey V, Fletcher LA, Forde CG, Gharib AM, Guo J, Howard R, Joseph PV, McGehee S, Ouwerkerk R, Raisinger K, Rozga I, Stagliano M, Walter M, Walter PJ, Yang S, Zhou M. Ultra-Processed Diets Cause Excess Calorie Intake and Weight Gain: An Inpatient Randomized Controlled Trial of Ad Libitum Food Intake. Cell Metab. 2019 Jul 2;30(1):67-77. e3. doi: 10.1016/j.cmet.2019.05.008. Epub 2019 May 16.
23. Gore AC, Chappell VA, Fenton SE, Flaws JA, Nadal A, Prins GS, Toppari J, Zoeller RT. EDC-2: the Endocrine Society's second Scientific Statement on endocrine-disrupting chemicals. Endocr Rev 2015; 36(6): E1–E150.
24. Pacyga DC, Sathyanarayana S, Strakovsky RS. Dietary Predictors of Phthalate and Bisphenol Exposures in Pregnant Women. Adv Nutr. 2019 May 30. pii: nmz029. doi: 10.1093/advances/nmz029.
25. Rancière F, Lyons JG, Loh VHY, et al. Bisphenol A and the risk of cardiometabolic disorders: a systematic review with meta-analysis of the epidemiological evidence. Environ Health 2015; 14:46. doi:10.1186/s12940-015-0036-5
26. Radke EG, Galizia A, Thayer KA, Cooper GS. Phthalate exposure and metabolic effects: a systematic review of the human epidemiological evidence. Environ Int. 2019 Nov; 132:104768. doi: 10.1016/j.envint.2019.04.040. Epub 2019 Jun 10.
27. Shoshtari-Yeganeh B, Zarean M, Mansourian M, Riahi R, Poursafa P, Teiri H, Rafiei N, Dehdashti B, Kelishadi R. Systematic review and meta-analysis on the association between phthalates exposure and insulin resistance. Environ Sci Pollut Res Int. 2019

Apr;26(10):9435-9442. doi: 10.1007/s11356-019-04373-1. Epub 2019 Feb 8. PMID: 30734259

28. Lopez-Espinosa MJ, Granada A, Araque P, Molina-Molina JM, Puertollano MC, Rivas A, Fernández M, Cerrillo I, Olea-Serrano MF, López C, Olea N. Oestrogenicity of paper and cardboard extracts used as food containers. Food Addit Contam. 2007 Jan;24(1):95-102.
29. Moreira MA, Andre LC, Cardeal ZL. Analysis of phthalate migration to food simulants in plastic containers during microwave operations. Int J Environ Res Public Health 2013; 11(1):507–26.
30. Buckley JP, Kim H, Wong E, Rebholz CM. Ultra-processed food consumption and exposure to phthalates and bisphenols in the US National Health and Nutrition Examination Survey, 2013-2014. Environ Int. 2019 Oct; 131:105057. doi: 10.1016/j.envint.2019.105057. Epub 2019 Aug 6.
31. National Health and Nutrition Examination Survey: Analytic Guidelines, 2011-2014 and 2015-2016. December 14, 2018.
32. NHANES. MEC In-Person Dietary Interviewers Procedures Manual. January 2009a. Available at: http://www.cdc.gov/nchs/data/nhanes/nhanes_09_10/DietaryInterviewers_Inperson.pdf
33. NHANES. Phone Follow-Up Dietary Interviewer Procedures Manual. September 2009b. Available at: http://www.cdc.gov/nchs/data/nhanes/nhanes_09_10/Dietary_PFU_09.pdf.
34. Moshfegh AJ, Rhodes DG, Baer DJ, Murayi T, Clemens JC, Rumpler WV, Paul DR, Sebastian RS, Kuczynski KC, Ingwersen LA, Staples RC, Cleveland LC. The USDA Automated Multiple-Pass Method reduces bias in the collection of energy intakes. Am J Clin Nutr 2008; 88:324-332.
35. Blanton CA, Moshfegh AJ, Baer DJ, Kretsch MJ. The USDA Automated Multiple-Pass Method accurately estimates group total energy and nutrient intake. J Nutr 2006 Oct; 136(10):2594-9.
36. Rumpler WV, Kramer M, Rhodes DG, Moshfegh AJ, Paul DR, Kramer M. Identifying sources of reporting error using measured food intake. Eur J Clin Nutr 2008; 62:544-52.
37. Automated Multiple-Pass Method. United States Department of Agriculture. Agriculture Research Service. http://www.ars.usda.gov/ba/bhnrc/fsrg (accessed August 2019).
38. National Health and Nutrition Examination Survey. NHANES Response Rates and Population Totals. Response Rates (2009-2016). <https://wwwn.cdc.gov/nchs/nhanes/ResponseRates.aspx> (accessed August 2019).
39. Calafat AM, Longnecker MP, Koch HM, Swan SH, Hauser R, Goldman LR, Lanphear BP, Rudel RA, Engel SM, Teitelbaum SL, et al. Optimal exposure biomarkers for nonpersistent chemicals in environmental epidemiology. Environ Health Perspect 2015;123(7): A166–8.
40. Johns LE, Cooper GS, Galizia A, Meeker JD. Exposure assessment issues in epidemiology studies of phthalates. Environ Int. 2015 Dec; 85:27-39. doi: 10.1016/j.envint.2015.08.005. Epub 2015 Aug 24
41. Centers for Disease Control and Prevention. National Center for Health Statistics. National Health and Nutrition Examination Survey. Laboratory Procedure Manual. Metabolites of phthalates and phthalate alternatives. NHANES 2015-2016.

42. Centers for Disease Control and Prevention. National Center for Health Statistics. National Health and Nutrition Examination Survey. Laboratory Procedure Manual. Personal Care and Consumer Product Chemicals and Metabolites: benzophenone-3, bisphenol A, bisphenol F, bisphenol S, 2,4-dichlorophenol, 2,5-dichlorophenol, methyl-, ethyl-, propyl-, and butyl parabens, triclosan, and triclocarban. NHANES 2015-2016.
43. Zota AR, Calafat AM, Woodruff TJ. 2014. Temporal trends in phthalate exposures: findings from the National Health and Nutrition Examination Survey, 2001–2010. Environ Health Perspect 122:235–241, doi: 10.1289/ehp.1306681.
44. Braun JM, Smith KW, Williams PL, Calafat AM, Berry K, Ehrlich S, et al. Variability of urinary phthalate metabolite and bisphenol A concentrations before and during pregnancy. Environmental health perspectives. 2012; 120(5):739–45. [PubMed: 22262702]
45. Polinski KJ, Dabelea D, Hamman RF, Adgate JL, Calafat AM, Ye X, Starling AP. Distribution and predictors of urinary concentrations of phthalate metabolites and phenols among pregnant women in the Healthy Start Study. Environ Res. 2018 Apr; 162:308-317. doi: 10.1016/j.envres.2018.01.025. Epub 2018 Feb 4.
46. Laboratory Procedure Manual. Urinary Creatinine. University of Minnesota, January 2011. Available online: https://www.cdc.gov/nchs/data/nhanes/nhanes_09_10/ALB_CR_F_met_creatinine.pdf (accessed on 12 February 2017).
47. Monteiro CA, Cannon G, Moubarac JC, Levy RB, Louzada ML, Jaime PC. The UN Decade of Nutrition, the NOVA food classification and the trouble with ultra-processing. Public Health Nutr 2018; 21:5-17.
48. Martinez Steele E, Baraldi LG, Louzada ML, Moubarac JC, Mozaffarian D, Monteiro CA. Ultra-processed foods and added sugars in the US diet: evidence from a nationally representative cross-sectional study. BMJ Open. 2016 Mar 9;6(3):e009892. doi: 10.1136/bmjopen-2015-009892.
49. Juul F, Martinez-Steele E, Parekh N, Monteiro CA, Chang VW. Ultraprocessed food consumption and excess weight among US adults. Br J Nutr 2018; 120:90-100. doi:10.1017/S0007114518001046
50. U.S. Department of Agriculture, Agricultural Research Service. USDA Food and Nutrient Database for Dietary Studies 5.0, 2011-2012, 2013-2014 and 2015-2016. Food Surveys Research Group Home Page, http://www.ars.usda.gov/ba/bhnrc/fsrg
51. USDA National Nutrient Database for Standard Reference, Release 24, 26 and 28. Version Current: May 2016. Internet: http://www.ars.usda.gov/ba/bhnrc/ndl.
52. Centers for Disease Control and Prevention. National Center for Health Statistics. National Health and Nutrition Examination Survey. Anthropometry procedures manuals: 2009-2010; 2011-2012; 2013-2014.
53. World Health Organization (2016) BMI classification. http://apps.who.int/bmi/index.jsp?introPage=intro_3.html (accessed July 2019).
54. Kuczmarski RJ, Ogden CL, Guo SS, et al. 2000 CDC growth charts for the United States: Methods and development. National Center for Health Statistics. Vital Health Stat 11(246). 2002 https://www.cdc.gov/nchs/data/series/sr_11/sr11_246.pdf (accessed July 2019).
55. U.S. Department of Health and Human Services. 2008 Physical Activity Guidelines for Americans. Washington (DC): U.S. Department of Health and Human Services; 2008. ODPHP Publication No. U0036. Available at: http://www.health.gov/paguidelines. Accessed April 2018.

56. US Department of Health and Human Services & US Department of Agriculture (2015) 2015–2020 Dietary Guidelines for Americans, 8th ed http://health.gov/dietaryguidelines/2015/guidelines (accessed September 2017).
57. Fats and fatty acids in human nutrition. Report of an expert consultation. FAO Food and Nutrition Paper (91). Rome, 2010.
58. Harrell, F. E., Jr. 2001. Regression Modeling Strategies: With Applications to Linear Models, Logistic Regression, and Survival Analysis. New York: Springer.
59. Desquilbet L, Mariotti F. Dose-response analyses using restricted cubic spline functions in public health research. Stat Med. 2010 Apr 30;29(9):1037-57. doi: 10.1002/sim.3841. Epub 2010 Jan 19.
60. Serrano SE, Braun J, Trasande L, Dills R, Sathyanarayana S. Phthalates and diet: a review of the food monitoring and epidemiology data. Environ Health 2014;13(1):43.
61. Cao XL. Phthalate esters in foods: sources, occurrence, and analytical methods. Compr Rev Food Sci Food Saf 2010;9(1):21–43.
62. Zota AR, Phillips CA, Mitro SD. Recent fast food consumption and bisphenol A and phthalates exposures among the U.S. population in NHANES, 2003–2010. Environ Health Perspect 2016;124(10):1521–8.
63. Watkins DJ, Eliot M, Sathyanarayana S, et al. Variability and Predictors of Urinary Concentrations of Phthalate Metabolites during Early Childhood. Environ Sci Technol. 2014; 48(15):8881–8890. DOI: 10.1021/es501744v [PubMed: 24977926]
64. Barr DB, Wilder LC, Caudill SP, Gonzalez AJ, Needham LL, Pirkle JL. Urinary creatinine concentrations in the U.S. population: implications for urinary biologic monitoring measurements. Environ Health Perspect. 2005 Feb;113(2):192-200.
65. NTP (National Toxicology Program). 2016. Report on Carcinogens, Fourteenth Edition.; Research Triangle Park, NC: U.S. Department of Health and Human Services, Public Health Service. https://ntp.niehs.nih.gov/go/roc14 (EndNote XML)
66. Geens T, Aerts D, Berthot C, Bourguignon JP, Goeyens L, Lecomte P, et al. 2012. A review of dietary and non-dietary exposure to bisphenol-A. Food Chem Toxicol 50:3725–3740.
67. Cao, X.L., Perez-Locas, C., Dufresne, G., Clement, G., Popovica, S., Beraldin, F., Dabeke, R.W., Feeley, M., 2011. Concentrations of bisphenol A in the composite food samples from the 2008 Canadian total diet study in Quebec City and dietary intake estimates. Food Addit. Contam. Part A. 28, 791–798.
68. Cao XL, Kosarac I, Popovic S, Zhou S, Smith D, Dabeka R. LC-MS/MS analysis of bisphenol S and five other bisphenols in total diet food samples. Food Addit Contam Part A Chem Anal Control Expo Risk Assess. 2019 Jul 30:1-8. doi: 10.1080/19440049.2019.1643042.
69. ECHA (European Chemicals Agency). Evaluation of New Scientific Evidence Concerning DINP and DIDP: In Relation to Entry 52 of Annex XVII to REACH Regulation (EC) No 1907/2006. 2012. https://echa.europa.eu/documents/10162/31b4067e-de40-4044-93e8-9c9ff1960715
70. Prentice, R.L., Mossavar-Rahmani, Y., Huang, Y., Van Horn, L., Beresford, S.A.A., Caan, B., et al., 2011. Evaluation and comparison of food records, recalls, and frequencies for energy and protein assessment by using recovery biomarkers. Am. J. Epidemiol. (5), 591–603 Sep 1.
71. Slining, M.M., Yoon, E.F., Davis, J., Hollingsworth, B., Miles, D., Ng, S.W., 2015. An approach to monitor food and nutrition from “factory to fork”. J. Acad. Nutr. Diet. 115 (1), 40–49.

Table 1. Study flowchart

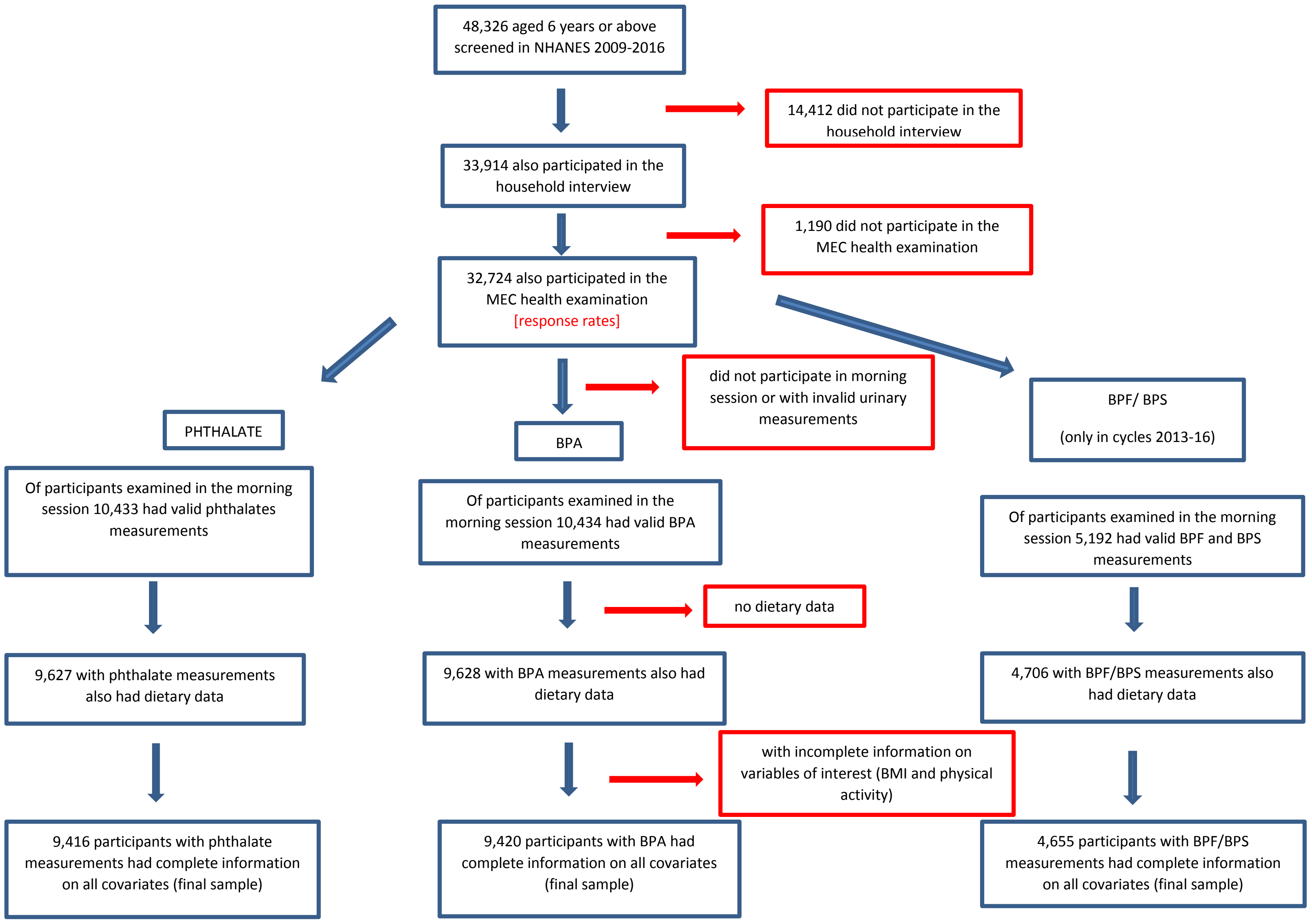

Table 2. Dietary contribution of ultra-processed foods and Phthalate/Bisphenol levels standardized (ηmol/g creatinine) according to characteristics of respondents. US population aged 6 and above (NHANES 2009-2016)

		Dietary contribution of ultra-processed foods (% total energy) (n=9,416)	PHTALATES (ηmol/g creatinine)					BISPHENOL (ηmol/g creatinine)[c]		
			ΣDEHP (n=9,416)	ΣDiNP (n=9,416)	mCNP (n=9,416)	mCPP (n=9,416)	mBzP (n=9,416)	BPA (n=9,420)	BPF (n=4,655)	BPS (n=4,655)
		mean (SE)	GM[a] (GSE)	GM (GSE)	GM (GSE)	GM (GSE)	GM (GSE)	GM (GSE)	GM (GSE)	GM (GSE)
**Gender**	**Men**	58.4 (0.4)	87.8 (1.0)	49.7 (1.0)	7.1 (1.0)	8.5 (1.0)	17.5 (1.0)	5.9 (1.0)	2.1 (1.1)	1.6 (1.1)
	**Women**	58.2 (0.5)	103.6 (1.0)£	56.5 (1.0)£	8.0 (1.0)£	9.3 (1.0)£	22.2 (1.0)£	7.0 (1.0)£	2.4 (1.0)£	1.9 (1.0)
**Age groups (years)**	**6 to 11**	68.2 (0.5)	176.5 (1.0)	78.9 (1.1)	11.7 (1.0)	16.6 (1.0)	50.8 (1.0)	8.8 (1.0)	2.3 (1.1)[A]	1.9 (1.0)
	**12 to 19**	66.9 (0.7)	90.5 (1.0)	52.1 (1.1)	6.8 (1.0)	8.4 (1.1)	23.8 (1.0)	5.6 (1.0)	2.0 (1.1)[A]	1.2 (1.0)
	**20 or above**	55.9 (0.4)*	90.3 (1.0)*	51.0 (1.0)*	7.3 (1.0)*	8.4 (1.0)*	17.5 (1.0)*	6.4 (1.0)*	2.3 (1.0)[A]	1.9 (1.0)*
**Race/ethnicity[b]**	**Mexican American**	56.8 (0.6)[A]	112.9 (1.1)[B]	47.7 (1.1)[AB]	6.6 (1.0)[A]	7.7 (1.0)[A]	20.8 (1.1)[AB]	6.4 (1.0)[A]	1.7 (1.1)[A]	2.3 (1.1)[B]
	**Other Hispanic**	53.5 (0.9)[B]	101.0 (1.0)[AB]	58.9 (1.1)[B]	7.0 (1.0)[A]	8.9 (1.1)[AB]	18.9 (1.1)[AB]	6.4 (1.1)[A]	1.7 (1.1)[A]	2.3 (1.1)[B]
	**Non-Hispanic White**	59.6 (0.5)[C]	94.2 (1.0)[A]	57.2 (1.1)[B]	8.1 (1.0)[B]	9.7 (1.0)[B]	19.8 (1.0)[AB]	6.5 (1.0)[A]	2.5 (1.1)[B]	1.6 (1.0)[A]
	**Non-Hispanic Black**	61.4 (0.8)[C]	84.8 (1.0)[C]	41.6 (1.1)[A]	6.4 (1.0)[A]	7.1 (1.1)[A]	21.5 (1.0)[B]	6.4 (1.0)[A]	2.1 (1.1)[AB]	2.0 (1.0)[B]
	**Other Race (including Multi-Racial)**	48.6 (1.0)[D]	99.4 (1.0)[AB]	43.8 (1.1)[A]	6.1 (1.0)[A]	7.4 (1.1)[A]	17.0 (1.1)[A]	5.9 (1.0)[A]	2.0 (1.1)[AB]	1.8 (1.1)[AB]
**Income to poverty[b]**	**0.00–1.30**	60.5 (0.7)[C]	103.5 (1.0)[B]	49.4 (1.0)[A]	6.9 (1.0)[A]	8.9 (1.0)[AB]	26.9 (1.0)[A]	7.0 (1.0)[B]	2.0 (1.0)[A]	2.0 (1.1)[B]
	**>1.30–3.50**	59.5 (0.7)[BC]	92.7 (1.0)[A]	50.0 (1.0)[A]	7.4 (1.0)[B]	8.7 (1.0)[AB]	21.4 (1.0)[B]	6.7 (1.0)[B]	2.4 (1.1)[A]	1.8 (1.1)[AB]
	**>3.50 and above**	56.3 (0.6)[A]	92.7 (1.0)[A]	60.3 (1.1)[B]	8.1 (1.0)[C]	9.3 (1.1)[B]	15.3 (1.0)[C]	6.0 (1.0)[A]	2.4 (1.1)[A]	1.6 (1.1)[A]
	**missing**	56.2 (1.2)[AB]	98.9 (1.1)[AB]	45.0 (1.1)[A]	7.1 (1.1)[ABC]	7.6 (1.1)[A]	19.6 (1.1)[B]	6.2 (1.1)[AB]	1.9 (1.1)[A]	2.1 (1.1)[B]
**Cycle**	**2009-10**	58.3 (0.8)	156.1 (1.1)	47.5 (1.1)	8.9 (1.0)	12.7 (1.1)	26.3 (1.1)	8.5 (1.0)	_	_

	**2011-12**	60.1 (0.9)	107.7 (1.0)	77.3 (1.1)	8.4 (1.0)	13.5 (1.1)	20.0 (1.0)	7.5 (1.0)	_	_
	**2013-14**	58.5 (0.8)	77.8 (1.0)	73.1 (1.0)	8.3 (1.0)	8.5 (1.1)	17.4 (1.0)	5.7 (1.0)	2.7 (1.1)	1.7 (1.1)
	**2015-16**	56.4 (0.7)*	65.4 (1.0)*	30.1 (1.1)*	5.2 (1.0)*	4.5 (1.1)*	17.0 (1.1)*	4.8 (1.0)*	1.9 (1.0)$^{\pounds}$	1.9 (1.1)
**BMI[b]**	**underweight**	57.0 (1.5)^{AB}	92.7 (1.1)	53.9 (1.1)^{A}	7.6 (1.1)^{A}	9.0 (1.1)	20.4 (1.1)	7.2 (1.1)^{A}	3.0 (1.2)^{A}	1.8 (1.1)^{A}
	**normoweight**	59.3 (0.6)^{B}	102.9 (1.0)	53.8 (1.0)^{A}	7.8 (1.0)^{A}	9.6 (1.0)	22.0 (1.0)	6.6 (1.0)^{A}	2.1 (1.1)^{A}	1.7 (1.1)^{A}
	**overweight**	56.3 (0.6)^{A}	91.1 (1.0)	52.1 (1.1)^{A}	7.3 (1.0)^{A}	8.6 (1.0)	17.1 (1.0)	6.3 (1.0)^{A}	2.3 (1.1)^{A}	1.7 (1.1)^{A}
	**obesity**	59.2 (0.5)^{B}	92.9 (1.0)*	53.1 (1.0)^{A}	7.4 (1.0)^{A}	8.5 (1.0)*	20.2 (1.0)*	6.4 (1.0)^{A}	2.3 (1.1)^{A}	1.9 (1.1)^{A}
**Physical activity[b]**	**Low**	58.5 (0.6)	92.3 (1.0)^{A}	49.7 (1.0)^{A}	6.8 (1.0)	8.1 (1.0)	19.2 (1.0)^{A}	6.2 (1.0)^{A}	2.3 (1.1)^{A}	1.8 (1.1)^{A}
	**Medium**	58.3 (0.8)	102.7 (1.0)^{B}	58.2 (1.1)^{B}	8.2 (1.0)	9.8 (1.1)	19.9 (1.0)^{A}	6.6 (1.0)^{A}	2.2 (1.1)^{A}	1.8 (1.1)^{A}
	**High**	58.2 (0.5)	95.5 (1.0)^{AB}	53.8 (1.0)^{AB}	7.8 (1.0)*	9.2 (1.0)*	20.2 (1.0)^{A}	6.6 (1.0)^{A}	2.3 (1.1)^{A}	1.8 (1.0)^{A}
**Current smoker**	**no**	58.0 (0.4)	96.8 (1.0)	54.9 (1.0)	7.8 (1.0)	9.1 (1.0)	19.4 (1.0)	6.3 (1.0)	2.2 (1.0)	1.7 (1.0)
	**yes**	59.7 (0.9)$^{\pounds}$	89.3 (1.0)$^{\pounds}$	44.5 (1.1)$^{\pounds}$	6.4 (1.0)$^{\pounds}$	8.1 (1.0)$^{\pounds}$	22.1 (1.0)$^{\pounds}$	6.9 (1.0)$^{\pounds}$	2.8 (1.1)$^{\pounds}$	2.0 (1.1)$^{\pounds}$
**Energy above recommended**	**no**	57.1 (0.5)	92.4 (1.0)	52.2 (1.0)	7.3 (1.0)	8.7 (1.0)	19.5 (1.0)	6.3 (1.0)	2.2 (1.1)	1.7 (1.0)
	**yes**	60.3 (0.5)$^{\pounds}$	100.6 (1.0)$^{\pounds}$	54.4 (1.0)	7.8 (1.0)$^{\pounds}$	9.2 (1.0)$^{\pounds}$	20.3 (1.0)	6.6 (1.0)	2.3 (1.1)	1.9 (1.0)
**Total fat intake (% of total energy intake) above >30%**	**no**	54.6 (0.6)	97.8 (1.0)	49.5 (1.0)	7.1 (1.0)	8.7 (1.0)	20.2 (1.0)	6.7 (1.0)	2.1 (1.1)	1.8 (1.1)
	**yes**	60.1 (0.4)$^{\pounds}$	94.4 (1.0)	54.9 (1.0)$^{\pounds}$	7.7 (1.0)$^{\pounds}$	9.0 (1.0)	19.6 (1.0)	6.3 (1.0)$^{\pounds}$	2.3 (1.0)	1.8 (1.0)
**Fat in UPF derived total energy intake (% of total energy intake) above median (18.7%)**	**no**	44.7 (0.4)	94.2 (1.0)	47.5 (1.0)	7.0 (1.0)	8.2 (1.0)	18.9 (1.0)	6.2 (1.0)	2.0 (1.0)	1.9 (1.0)
	**yes**	71.9 (0.3)$^{\pounds}$	96.8 (1.0)	59.3 (1.0)$^{\pounds}$	8.0 (1.0)$^{\pounds}$	9.6 (1.0)$^{\pounds}$	20.7 (1.0)$^{\pounds}$	6.6 (1.0)$^{\pounds}$	2.5 (1.1)$^{\pounds}$	1.7 (1.0)$^{\pounds}$
**Total**		58.3 (0.4)	95.5 (1.0)	53.1 (1.0)	7.5 (1.0)	8.9 (1.0)	19.8 (1.0)	6.4 (1.0)	2.3 (1.0)	1.8 (1.0)

[a]GM= Geometric means; GSE= Geometric standard error

[b]Values sharing a letter in the group label are not significantly different at the p<0.05 level (using Bonferroni inequality adjustment for multiple comparisons).

[c]Bisphenol F and S analysis were only measured in 2013-2016 cycles

*Statistically significant linear trend (p<0.05)

£Statistically significant (p<0.05)

Table 3. Phthalate/Bisphenol levels according to the quintiles of the dietary share of ultra-processed foods. Subsample of US population aged 6 + years (NHANES 2009-2016)

		Quintile of dietary share of ultra-processed foods (% of total energy intake)[a]					
		Q1	Q2	Q3	Q4	Q5	*p for trend*
ΣDEHP (GM[b])	Crude (ηmol/mL)	0.08	0.09	0.09	0.09	0.10	<0.001
	Standardized (ηmol/g creatinine)	93.3	96.2	91.9	96.5	100.0	0.075
	Adjusted for socio-demographic variables (ηmol/g creat)[c]	97.9	96.5	91.7	95.5	96.0	0.493
	Adjusted for socio-demographic + other variables (ηmol/g creat)[d]	98.4	96.4	91.5	95.3	96.0	0.425
ΣDiNP (GM)	Crude (ηmol/mL)	0.04	0.05	0.05	0.06	0.06	<0.001
	Standardized (ηmol/g creatinine)	44.5	52.1	51.3	58.0	61.0	<0.001
	Adjusted for socio-demographic variables (ηmol/g creat)[c]	47.0	52.5	50.7	58.3	57.7	0.001
	Adjusted for socio-demographic + other variables (ηmol/g creat)[d]	46.9	52.4	50.6	58.2	57.9	0.001
mCNP (GM)	Crude (ηmol/mL)	0.006	0.007	0.007	0.008	0.008	<0.001
	Standardized (ηmol/g creatinine)	6.5	7.4	7.4	8.1	8.1	<0.001
	Adjusted for socio-demographic variables (ηmol/g creat)[c]	6.9	7.5	7.3	8.1	7.8	0.001
	Adjusted for socio-demographic + other variables (ηmol/g creat)[d]	6.9	7.4	7.3	8.1	7.9	0.001
mCPP (GM)	Crude (ηmol/mL)	0.007	0.008	0.008	0.009	0.011	<0.001
	Standardized (ηmol/g creatinine)	7.8	8.9	8.2	9.7	10.1	<0.001
	Adjusted for socio-demographic variables (ηmol/g creat)[c]	8.4	9.1	8.1	9.5	9.4	0.039
	Adjusted for socio-demographic + other variables (ηmol/g creat)[d]	8.4	9.1	8.1	9.5	9.4	0.035
mBzP (GM)	Crude (ηmol/mL)	0.02	0.02	0.02	0.02	0.03	<0.001
	Standardized (ηmol/g creatinine)	17.3	17.8	19.1	21.6	24.1	<0.001
	Adjusted for socio-demographic variables (ηmol/g creat)[c]	19.1	18.7	19.3	20.6	21.5	0.007

	Adjusted for socio-demographic + other variables (ηmol/g creat)[d]	19.2	18.8	19.3	20.5	21.3	0.017
BPA (GM)	Crude (ηmol/mL)	0.005	0.006	0.006	0.007	0.007	<0.001
	Standardized (ηmol/g creatinine)	6.1	6.1	6.3	6.7	6.9	0.001
	Adjusted for socio-demographic variables (ηmol/g creat)[c]	6.3	6.2	6.3	6.7	6.7	0.046
	Adjusted for socio-demographic + other variables (ηmol/g creat)[d]	6.3	6.2	6.3	6.7	6.7	0.056
BPF (GM)	Crude (ηmol/mL)	0.002	0.002	0.002	0.003	0.003	<0.001
	Standardized (ηmol/g creatinine)	1.8	2.3	2.3	2.5	2.4	0.001
	Adjusted for socio-demographic variables (ηmol/g creat)[c]	1.8	2.3	2.3	2.6	2.4	0.003
	Adjusted for socio-demographic + other variables (ηmol/g creat)[d]	1.8	2.3	2.3	2.5	2.5	0.004
BPS (GM)	Crude (ηmol/mL)	0.0020	0.0017	0.0017	0.0017	0.0018	0.108
	Standardized (ηmol/g creatinine)	2.2	1.8	1.8	1.7	1.6	<0.001
	Adjusted for socio-demographic variables (ηmol/g creat)[c]	2.1	1.8	1.8	1.7	1.6	0.002
	Adjusted for socio-demographic + other variables (ηmol/g creat)[d]	2.1	1.8	1.8	1.7	1.6	0.001

[a]For all phthalates: Mean (range) dietary share of ultra-processed foods per quintile: 1st=27.1 (0 to 39.5); 2nd= 46.8 (39.5 to 53.3); 3rd= 59.3 (53.3 to 65.2); 4th=71.1 (65.2 to 77.7); 5th= 87.3 (77.7 to 100)

bGeometric means (GM) presented in all cases

[c]Adjusted for cycle, sex, age group (6 to 11, 12 to 19, +20), race/ethnicity (Mexican American, Other Hispanic, Non-Hispanic White, Non-Hispanic Black, Other Race), ratio of family income to poverty (Supplemental Nutrition Assistance Program 0.00–1.30, >1.30–3.50 and >3.50 and over).

[d]Aditionally adjusted for energy intake above recommended levels (y/n), BMI (underweight, normoweight, overweight, obesity), physical activity (low, medium, high) and current smoking (y/n).

Table 4. Phthalate/Bisphenol levels according to tertiles of dietary share of ultra-processed ready-to-heat and all remaining subgroupsa. Subsample of US population aged 6 + years (NHANES 2009-2016)

Tertiles of ultra-processed food subgroups (% of total energy)		PHTHALATES (ηmol/g creatinine)					BISPHENOL (ηmol/g creatinine)		
		ΣDEHP	ΣDiNP	mCNP	mCPP	mBzP	BPA	BPF	BPS
**Ready-to-heat[a]**	**T1**	94.6	47.1	7.1	8.4	19.2	6.3	2.1	1.9
	**T2**	99.6	58.0	7.4	9.0	20.3	6.4	2.7	1.6
	**T3**	95.5	62.4	8.2	9.8	20.6	6.7	2.3	1.7
	**p for trend**	0.644	<0.001	<0.001	<0.001	<0.001	0.042	0.164	0.056
**Remaining subgroups[b]**	**T1**	97.6	53.6	7.4	9.0	19.9	6.3	1.9	2.0
	**T2**	94.6	53.4	7.7	9.0	19.1	6.6	2.5	1.7
	**T3**	94.4	52.2	7.5	8.7	20.4	6.4	2.4	1.6
	**p for trend**	0.208	0.573	0.586	0.364	0.487	0.525	0.005	<0.001

Note: All models adjusted for sex, age group (6 to 11, 12 to 19, +20), race/ethnicity (Mexican American, Other Hispanic, Non-Hispanic White, Non-Hispanic Black, Other Race), ratio of family income to poverty (Supplemental Nutrition Assistance Program 0.00–1.30, >1.30–3.50 and >3.50 and over)

[a]For all phthalates: Mean (range) dietary share of ready-to-heat ultra-processed foods per tertile: 1st=0 (0 to 0); 2nd= 6.5 (0.1 to 11.4); 3rd= 30.7 (11.4 to 100)

[b]For all phthalates: Mean (range) dietary share of remaining ultra-processed foods per tertile: 1st=25.9 (0 to 38.7); 2nd=47.3 (38.7 to 55.8); 3rd=68.9 (55.8 to 100)

Click here to access/download
Supporting Information
Phthalates_supplementary_20200131.docx
