# Supplementary material for: Association between dietary contribution of ultra-processed foods and urinary concentrations of phthalates and bisphenol in a nationally representative sample of the US population aged 6 years and older
Source: PLoS One. 2020 Jul 31;15(7):e0236738. doi: 10.1371/journal.pone.0236738 (PMC7394369; doi:10.1371/journal.pone.0236738)
Supplement: Attachment — Submitted filename: Response_to_reviewers.docx [file pone.0236738.s013.docx]

**However, the title of the manuscript submitted requires some minor changes. It is too general to state "US sample", instead of the "US population aged 6 years and older" in the title of the manuscript.**

We thank you for the suggestion. We have changed the title as suggested and now reads:

“Association between dietary contribution of ultra-processed foods and urinary concentrations of phthalates and bisphenol in a nationally representative sample of the US population aged 6 years and older”.
